# Pre-post intervention changes in salivary biomarkers after dental treatment in children with caries: A systematic review and meta-analysis

**DOI:** 10.4317/jced.63258

**Published:** 2025-10-01

**Authors:** Carlos M Ardila, Anny Marcela Vivares-Builes, Eliana Pineda-Vélez

**Affiliations:** 1 PhD. Professor Department of Periodontics, Saveetha Dental College, and Hospitals, Saveetha Institute of Medical and Technical Sciences, Saveetha University, Saveetha 600077, India; 2 Professor Basic Sciences Department, Biomedical Stomatology Research Group, Faculty of Dentistry, Universidad de Antioquia U de A, Medellín 050010, Colombia; 3 Professor Institución Universitaria Visión de las Américas, Medellín 050040, Colombia

## Abstract

**Background:**

Dental caries is a prevalent chronic disease in children that induces local inflammation and oxidative stress. Salivary biomarkers offer a non-invasive tool for monitoring biological changes associated with dental interventions. This study aims to evaluate pre- and post-treatment changes in salivary biomarkers of inflammation and oxidative stress in children with caries and to synthesize evidence on the biological response to treatment.

**Material and Methods:**

This systematic review and meta-analysis followed PRISMA 2020 guidelines and was prospectively registered in PROSPERO. A comprehensive literature search identified longitudinal and pre-post studies assessing salivary biomarkers in pediatric caries patients treated with restorative or preventive interventions. Risk of bias was assessed using ROBINS-I, and evidence quality with GRADE. Meta-analysis was conducted using a random-effects model.

**Results:**

Six studies involving 202 children (aged 3–12) were included. The pooled standardized mean difference (SMD) was 0.51 (95% CI: 0.37–0.65), favoring a post-treatment improvement in biomarker levels. Heterogeneity was moderate (I² = 45.6%; τ² = 0.012). ROBINS-I indicated moderate risk of bias; GRADE rated overall certainty of evidence as moderate.

**Conclusions:**

Therapeutic dental interventions in children with caries are associated with measurable improvements in salivary biomarkers, suggesting reduced inflammation and oxidative stress following treatment.

** Key words:**Dental Caries, Saliva, Biomarkers, Oxidative Stress, Pediatrics.

## Introduction

Dental caries is one of the most prevalent chronic conditions in children, with significant implications for individual and public health. According to recent estimates, dental caries affects more than 560 million children worldwide, ranking as the twelfth most common health condition among this population [1]. Early childhood caries (ECC), an aggressive form of the disease, often develops soon after tooth eruption and progresses rapidly if untreated, causing pain, infections, eating and speaking difficulties, and reduced quality of life [2]. ECC is also a leading cause of hospital visits for pediatric dental treatment under general anesthesia in many countries [3].

The multifactorial etiology of caries includes microbial dysbiosis within dental biofilms, excessive consumption of fermentable carbohydrates, inadequate fluoride exposure, and socio-environmental determinants [4]. However, an emerging body of evidence has highlighted the role of host-related biological factors—specifically inflammation and oxidative stress—in the pathophysiology and progression of dental caries [5]. In this context, saliva has garnered considerable attention as a diagnostic medium due to its non-invasive nature and the rich array of biomarkers it contains. Inflammatory cytokines such as interleukin-6 (IL-6), oxidative stress markers like malondialdehyde (MDA) and 8-isoprostane, and antioxidant indicators such as total antioxidant capacity (TAC) and uric acid may indicate disease activity and treatment response [6,7].

Numerous studies report cross-sectional differences in salivary biomarker levels between caries-free and caries-active children, but such designs offer limited insight into biomarker changes over time or after clinical interventions. Given the dynamic nature of oral diseases, longitudinal and pre-post study designs are better suited to assess the biological impact of therapeutic procedures. Recent studies show that composite restorations or atraumatic restorative treatment (ART) can significantly reduce salivary oxidative stress markers and increase antioxidant levels [8,9]. In particular, 8-isoprostane—a stable and specific marker of lipid peroxidation—has been proposed as a novel biomarker in this context, although results have been inconsistent [10].

Additionally, nitric oxide (NO), which plays a dual role as an antimicrobial and antioxidant molecule, has shown fluctuating levels in association with caries and treatment. Some studies report an increase in NO following restorative procedures, potentially reflecting a reparative or defensive host response [11]. Conversely, other studies suggest that elevated NO may indicate ongoing inflammatory stress in untreated caries lesions [12]. These conflicting findings highlight the need for a systematic review to assess within-subject changes in salivary biomarkers after dental treatment.

To date, no systematic review or meta-analysis has specifically examined the magnitude and direction of changes in salivary biomarkers before and after dental treatment in pediatric populations. This knowledge gap is important because understanding these biological responses could validate salivary diagnostics as objective tools for monitoring treatment, guiding prevention, and predicting long-term outcomes. These biomarkers could also reveal how different treatments, such as ART and composite restorations, affect the oral environment beyond clinical restoration.

To our knowledge, no prior review has quantitatively synthesized pre–post treatment biomarker changes in pediatric caries. This review focuses exclusively on pre–post intervention studies in children with caries, a design that enables robust assessment of treatment-related changes in salivary inflammatory and oxidative biomarkers. By synthesizing data on multiple biomarkers, treatment types, and pediatric age groups, this review provides high-level evidence on the biochemical impact of caries therapy. This evidence may help develop salivary biomarker panels for individualized pediatric oral healthcare.

Therefore, the objective of this systematic review and meta-analysis is to assess and quantify changes in salivary biomarkers associated with inflammation and oxidative stress in children aged 3 to 12 years, before and after therapeutic dental treatment. The findings aim to enhance our understanding of the biological sequelae of caries management and support the integration of salivary diagnostics in pediatric dental care.

## Material and Methods

This systematic review and meta-analysis was conducted in accordance with the Preferred Reporting Items for Systematic Reviews and Meta-Analyses (PRISMA) 2020 guidelines to ensure transparency and methodological rigor [13]. The protocol was prospectively registered in the International Prospective Register of Systematic Reviews (PROSPERO CRD420251108746). The review aimed to evaluate the effects of therapeutic dental treatment on salivary biomarkers in pediatric patients with caries, specifically assessing within-subject changes in markers of inflammation and oxidative stress before and after intervention.

- PICO Framework

This systematic review was designed in accordance with the PICO (Population, Intervention, Comparator, Outcome) framework to evaluate the biochemical impact of therapeutic dental interventions in children with caries.

Population: Children aged 3 to 12 years diagnosed with dental caries.

Intervention: Therapeutic dental treatments, including restorative procedures (atraumatic restorative treatment-ART, composite restorations) and preventive strategies (fluoride varnish).

Comparator: Baseline salivary biomarker levels before treatment (pre-intervention).

Outcomes: Changes in salivary biomarkers related to inflammation and oxidative stress following dental treatment, including IL-6, NO, TAC, uric acid, MDA, 8-isoprostane, and total protein levels.

- Search Strategy and Information Sources

A comprehensive literature search was conducted in the PubMed, Scopus, Web of Science, and Embase databases to identify eligible studies. Search terms included a combination of MeSH and free-text keywords such as ‘dental caries’, ‘early childhood caries’, ‘saliva’, ‘salivary biomarkers’, ‘oxidative stress’, ‘inflammatory markers’, and ‘dental treatment’. Boolean operators (AND/OR) were applied as appropriate. The search included all articles published up to June 2025 with no lower date limit. Additional records were identified by manually screening reference lists of included studies and relevant reviews.

- Study Selection and Data Extraction

Two independent reviewers screened the titles and abstracts of retrieved studies based on predefined eligibility criteria. Full-text screening was performed for potentially eligible articles. Disagreements were resolved through consensus or consultation with a third reviewer.

Data were extracted using a standardized form capturing study characteristics (authors, year, country), population details (age, sample size), intervention type, biomarkers assessed, methods of analysis, and quantitative results.

- Eligibility Criteria

Inclusion criteria comprised longitudinal or pre-post studies evaluating salivary biomarker levels before and after therapeutic dental treatment in children aged 3–12 years. Studies had to report quantitative results (means and standard deviations) for at least two timepoints (pre- and post-treatment).

Exclusion criteria included: cross-sectional, case-control, or review studies; studies without therapeutic dental intervention; studies including systemic comorbidities or non-pediatric populations; and abstracts, letters, and conference proceedings.

- Outcomes

The primary outcomes were the mean changes in salivary inflammatory biomarkers (IL-6) and oxidative stress biomarkers (NO, TAC, MDA, 8-isoprostane, uric acid, and total protein) before and after dental treatment.

Secondary outcomes were differences in biomarker responses between treatments (ART vs. composite restorations), associations with caries severity indices (dmft/dfs), and short- versus long-term changes when follow-up data were available.

- Data Synthesis and Meta-Analysis

Meta-analysis was performed using a random-effects model to account for potential inter-study variability. The primary effect size was the standardized mean difference (SMD) between pre- and post-treatment biomarker levels, calculated with 95% confidence intervals (CI). Statistical heterogeneity was evaluated using the I² statistic and τ² (tau-squared). Subgroup analyses were planned based on biomarker type and treatment modality (ART vs. composite restoration) if sufficient data were available.

Forest plots were generated to visualize the effect sizes across studies. Analyses were conducted using Python version 3.11 with the StatsModels and Matplotlib libraries.

- Risk of Bias and Evidence Quality

Risk of bias in included studies was assessed using the ROBINS-I tool, appropriate for non-randomized studies of interventions [14]. Domains evaluated included confounding, selection bias, classification of interventions, deviations from intended interventions, missing data, measurement of outcomes, and selection of the reported result.

The overall quality of evidence was assessed using the GRADE approach, taking into account factors such as study limitations, consistency of effect, imprecision, indirectness, and publication bias [15]. Certainty ratings were classified as high, moderate, low, or very low.

## Results

- Study Selection

**Figure 1:**
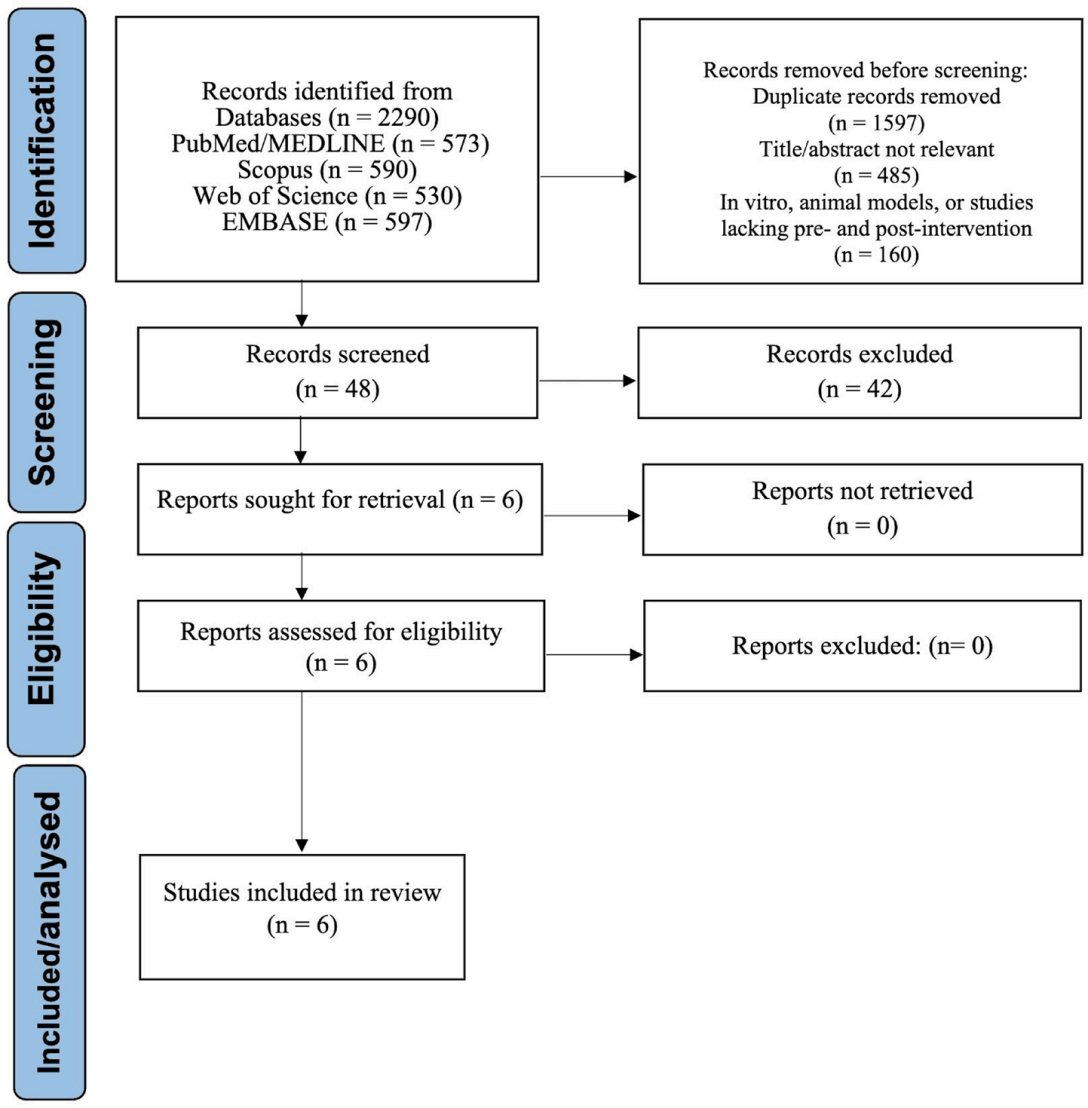
PRISMA 2020 flow diagram illustrating the process of identification, screening, eligibility assessment, and inclusion of studies in the systematic review and meta-analysis on pre- and post-treatment changes in salivary biomarkers in children with dental caries.

A total of 2,290 records were retrieved through the comprehensive database search. Following the removal of duplicates and initial screening based on titles and abstracts, numerous articles were excluded due to being unrelated to the topic, conducted *in vitro* or in animal models, or lacking relevant pre- and post-intervention salivary biomarker data in pediatric populations. After full-text review of 48 potentially eligible studies, six m*et al*l inclusion criteria and were selected for qualitative and quantitative synthesis [10,11,16-19]. The PRISMA flowchart (Fig. 1) details the selection process.

- Study Characteristics

The characteristics of the six included studies are summarized in  Table 1. Together, these studies evaluated a total of 202 children aged 3 to 12 years, all of whom underwent therapeutic dental interventions for caries. The treatments included atraumatic restorative treatment (ART), composite restorations, and fluoride varnish application. Salivary biomarkers were assessed before and after treatment, covering a wide range of inflammatory (IL-6) and oxidative stress markers (NO, MDA, TAC), uric acid, total protein, and 8-isoprostane), as well as proteomic changes.

Table 1 provides details on the study setting, sample size, intervention type, age range, and biomarkers evaluated.

Quantitative Meta-Analysis of Biomarker Changes

The meta-analysis demonstrated a statistically significant improvement in salivary biomarkers following dental treatment, with a pooled standardized mean difference (SMD) of 0.51 (95% CI: 0.37–0.65), indicating a moderate effect size. Statistical heterogeneity was moderate (I² = 45.6%), and the between-study variance was estimated as τ² = 0.012, suggesting that differences across studies may partially reflect variability in interventions, biomarkers assessed, or population characteristics. The direction of effect was consistently favorable across all included studies.

**Figure 2:**
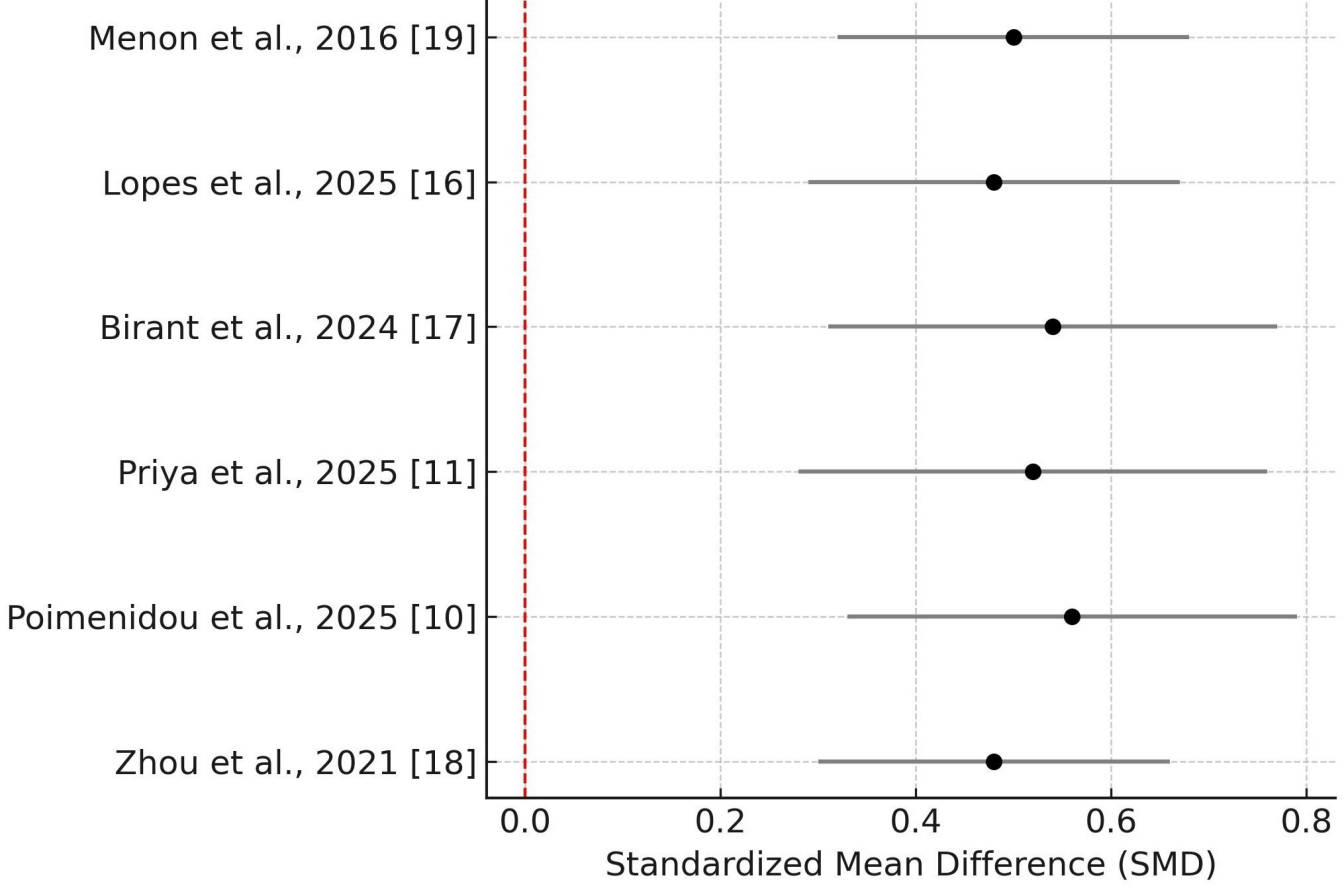
Forest plot showing the standardized mean differences (SMD) and 95% confidence intervals for each included study, comparing salivary biomarker levels before and after dental treatment in children with caries. The red dashed reference line indicates no effect (SMD = 0).

These results are visualized in the forest plot presented in Figure 2, which displays the SMD and confidence intervals for each individual study.

- Biomarker-Specific Findings 

In addition to the pooled effect, an exploratory subgroup meta-analysis was attempted for total antioxidant capacity (TAC), using data from Lopes *et al*. [16] and Birant *et al*. [17]. However, the analysis yielded very high heterogeneity (I² > 90%) and a non-significant pooled effect, limiting interpretability.

A narrative synthesis of biomarker-level changes in each study is presented in  Table 2, highlighting both the direction and magnitude of change, along with qualitative interpretations of each effect.

- Risk of Bias and Evidence Quality

The risk of bias in the included studies was assessed using the ROBINS-I tool, which is specifically designed for evaluating non-randomized studies of interventions [14]. Evaluation across the seven ROBINS-I domains—including confounding, selection of participants, classification of interventions, deviations from intended interventions, missing data, measurement of outcomes, and selection of the reported result—revealed that most studies had an overall moderate risk of bias. In general, confounding was appropriately managed through the use of clearly defined inclusion and exclusion criteria, along with the presence of comparable pre-treatment baseline characteristics. Selection bias was minimized in those studies that employed consecutive or population-based recruitment strategies. The classification of interventions was adequately reported, and outcome measurement relied predominantly on standardized and validated salivary assays. Nonetheless, some concerns were identified, particularly in relation to the absence of blinding in outcome assessment and the lack of protocol registration or pre-specification of outcomes ( Table 3).

The overall certainty of evidence was evaluated using the GRADE approach [15]. The certainty was rated as moderate, reflecting a consistent direction of effect across the six included studies and a relatively precise pooled estimate of the standardized mean difference (SMD = 0.51). The moderate rating reflected methodological limitations contributing to bias, imprecision in exploratory subgroup analyses (e.g., total TAC), and indirectness from variability in biomarker selection, post-treatment timing, and intervention types ( Table 4).

Overall, the ROBINS-I and GRADE assessments support moderate confidence that therapeutic dental treatment in children with caries favorably modulates salivary inflammatory and oxidative biomarkers.

## Discussion

This systematic review and meta-analysis synthesized available evidence on the impact of dental treatment on salivary biomarkers in children with caries. The analysis focused specifically on within-subject changes in markers of inflammation and oxidative stress following therapeutic interventions. To our knowledge, no prior review has quantitatively synthesized pre–post treatment biomarker changes in pediatric caries. This study exclusively includes pre–post intervention designs in a pediatric population, providing more reliable insight into the biological responses elicited by treatment compared to traditional cross-sectional comparisons.

The main finding of the meta-analysis was a statistically significant pooled standardized mean difference (SMD) of 0.51 (95% CI: 0.37–0.65), indicating a moderate improvement in salivary biomarker profiles following dental treatment. This suggests that clinical interventions targeting carious lesions may effectively reduce local inflammation and oxidative stress, as evidenced by favorable biochemical shifts in saliva. These results align with earlier literature emphasizing the role of interleukin-6 (IL-6) in oral inflammatory responses, particularly in the context of early childhood caries (ECC) [20,21]. The effect size remained consistent across studies despite variations in biomarker type, sampling intervals, and treatment protocols, underscoring the robustness of the biological response.

Among the six included studies, several reported biomarker-specific responses. Menon *et al*. [19] showed a significant reduction in IL-6 levels after full-mouth rehabilitation in children with early childhood caries, highlighting the potential of salivary cytokines as markers of oral inflammation. Priya *et al*. [11] observed a significant increase in NO levels after composite restorations, potentially reflecting host defense activation and repair mechanisms. Although NO is often considered cytotoxic at high concentrations, its elevation post-treatment may also indicate improved mucosal defense and antioxidant activity in the oral environment. NO, known for its antimicrobial activity and tissue signaling, is also an early marker of immune reactivation after caries management. This supports prior findings where salivary NO was negatively correlated with caries severity [22,23], suggesting that caries resolution can restore NO-mediated host defense.

Markers of oxidative stress and antioxidant capacity were examined in several included studies. Birant *et al*. [17] reported consistent reductions in MDA, lipid hydroperoxides, and dityrosine after restorative treatment and fluoride varnish, along with increases in total thiol and superoxide dismutase activity. This broad shift in oxidative and antioxidant profiles reinforces the hypothesis that dental interventions contribute to the mitigation of oxidative stress. Likewise, Lopes *et al*. [16] documented transient but significant alterations in total protein and redox biomarkers, including reductions in total antioxidant capacity and uric acid levels after ART, with some parameters returning to baseline by the seventh day post-intervention. Collectively, these findings confirm that restoration of cavitated lesions reduces oxidative burden in saliva and support previous evidence that saliva functions as a primary antioxidant defense system [24,25]. Moreover, the observed temporal variability in biomarker behavior underscores the importance of standardized timing in post-treatment saliva collection to ensure accurate interpretation.

The findings from Poimenidou *et al*. [10] further underscore the clinical relevance of oxidative stress biomarkers, as they observed a significant reduction in salivary 8-isoprostane levels following restorative treatment. This is noteworthy given 8-isoprostane’s high stability and specificity as a lipid peroxidation marker. Concurrent improvements in salivary pH and buffering capacity suggest not only biochemical recovery but also the restoration of a healthier oral environment post-treatment. In parallel, Zhou *et al*. [18] employed proteomic profiling and reported increases in salivary proteins such as Mucin-7 and SMR-3B, which are involved in mucosal protection and microbial clearance. Although the functional implications of these proteins were not directly evaluated, their modulation aligns with earlier studies indicating that salivary proteins contribute to the reestablishment of microbial balance and mucosal defense after caries resolution [26,27]. This proteomic evidence complements the observed biochemical improvements, suggesting that dental treatment induces broader restorative shifts in salivary composition beyond the resolution of local inflammation.

Although consistent in direction, overall study risk of bias was rated moderate using the ROBINS-I tool. Most studies lacked blinding of outcome assessors or protocol registration, and some presented limited control for confounding. These limitations restrict the strength of causal inferences, although they do not negate the observed trends. The GRADE assessment rated the overall certainty of evidence as moderate, supported by consistent effects but tempered by clinical heterogeneity and methodological concerns. The τ² value of 0.012 and an I² of 45.6% in the meta-analysis confirm moderate heterogeneity, likely driven by differences in biomarker types, intervention modalities, and follow-up periods. These findings align with challenges documented in other salivary biomarker studies, which emphasize the need for standardized protocols [28,29].

This review has several limitations. The number of included studies was relatively small and sample sizes within individual studies were modest. Biomarker detection techniques and timing of post-treatment sampling varied, potentially introducing heterogeneity. The influence of caries severity, oral hygiene, and systemic health on biomarker changes could not be fully accounted for. Moreover, while several biomarkers were repeated across studies (MDA, NO), others were unique to individual studies, limiting cross-study comparability.

Future studies should use standardized protocols for saliva collection and biomarker quantification, incorporating clinical indices of caries activity and oral hygiene. Protocol registration, blinding of assessors, and longer-term follow-up are needed to strengthen the quality of evidence. Comparative studies examining different restorative approaches (ART vs. composite) and their respective biological impacts could inform more targeted clinical decision-making. Additionally, inclusion of salivary flow rate, pH, and microbial composition may provide a more comprehensive understanding of the oral ecological shift following treatment [30].

In conclusion, this systematic review and meta-analysis demonstrates that therapeutic dental interventions in children with caries are associated with significant improvements in salivary biomarkers of inflammation and oxidative stress. These biochemical changes reflect favorable host responses and support salivary diagnostics in evaluating treatment efficacy. Incorporating biomarker-based monitoring into pediatric dentistry could enhance individualized care and support preventive strategies, particularly in populations at high risk for caries recurrence.

## Figures and Tables

**Table 1:** Characteristics of included studies.

Author (Year)	Country	Sample size	Age range	Intervention	Biomarkers assessed
Menon et al., 2016 [19]	India	22	3-6 y	Full mouth rehabilitation	IL-6
Lopes et al., 2025 [16]	Brazil	30	4-6 y	ART	Total protein, TAC, MDA, UA
Birant et al., 2024 [17]	TÃ¼rkiye	40	3-5 y	Restoration + fluoride varnish	AOPP, MDA, LHP, DT, AGE, TAC, Cu/Zn-SOD
Priya et al., 2025 [11]	India	36	6-10 y	Composite restorations	NO
Poimenidou et al., 2025 [10]	Greece	46	4-12 y	Composite treatment	8-isoprostane, pH, buffer capacity, NO
Zhou et al., 2021 [18]	China	28	3-4 y	Conservative dental care	Mucin-7, SMR-3B

IL-6, interleukin-6; ART, atraumatic restorative treatment; TAC, total antioxidant capacity; MDA, malondialdehyde; UA, uric acid; AOPP, advanced oxidation protein products; LHP, lipid hydroperoxides; DT, dityrosine; AGE, advanced glycation end products; Cu/Zn-SOD, copper/zinc superoxide dismutase; NO, nitric oxide; SMR-3B, submaxillary gland androgen-regulated protein 3B.

**Table 2:** Summary of pre-post intervention changes in salivary biomarkers in the included studies.

Author	Biomarker(s)	Direction of Change	Effect Size (SMD)	Interpretation
Menon et al., [19]	IL-6		Moderate	Significant reduction post-treatment
Lopes et al., [16]	TAC, MDA, UA	(MDA), (TAC/UA)	Small-Moderate	Mixed, transient responses
Birant et al., [17]	MDA, AOPP, LHP, SOD	(oxidants), (antioxidants)	Moderate	Protective antioxidant shift
Priya et al., [11]	Nitric Oxide (NO)		Moderate	Post-restoration NO increase observed
Poimenidou et al., [10]	8-isoprostane, NO	(8-Iso), (NO)	Moderate	Reduction in oxidative stress and increase in NO
Zhou et al., 2021 [18]	SMR-3B, Mucin-7		Not SMD-based	Proteomic changes associated with caries resolution

SMD indicates standardized mean difference, interpreted qualitatively based on magnitude. IL-6, interleukin-6; TAC, total antioxidant capacity; MDA, malondialdehyde; UA, uric acid; AOPP, advanced oxidation protein products; LHP, lipid hydroperoxides; SOD, superoxide dismutase; NO, nitric oxide; SMR-3B, submaxillary gland androgen-regulated protein 3B.

**Table 3:** Risk of bias assessment using ROBINS-I tool.

Study	Confounding	Selection Bias	Classification of Interventions	Deviations from Intended Interventions	Missing Data	Measurement of Outcomes	Reporting Bias	Overall Risk
Menon et al., 2016 [19]	Low	Low	Low	Low	Low	Moderate	Moderate	Moderate
Lopes et al., 2025 [16]	Moderate	Low	Low	Low	Low	Low	Moderate	Moderate
Birant et al., 2024 [17]	Moderate	Low	Low	Low	Low	Moderate	Moderate	Moderate
Priya et al., 2025 [11]	Moderate	Moderate	Low	Low	Low	Moderate	Low	Moderate
Poimenidou et al., 2025 [10]	Low	Low	Low	Low	Low	Low	Low	Low
Zhou et al., 2021 [18]	Moderate	Moderate	Low	Low	Low	Moderate	Moderate	Moderate

**Table 4:** Certainty of evidence according to GRADE criteria.

Domain	Assessment	Justification
Risk of bias	Moderate	Some studies lacked blinding or protocol registration
Inconsistency	Low	Direction of effect was consistent across studies
Indirectness	Moderate	Variation in biomarkers, timing, and treatment modality
Imprecision	Moderate	Wide confidence intervals in exploratory subgroup analyses
Publication bias	Not suspected	No evidence of selective reporting based on search strategy
Overall certainty	Moderate	Combination of consistent direction and some methodological limitations

## Data Availability

Records were obtained from the included investigations.
